# Resilient Sensor Networks with Spatiotemporal Interpolation of Missing Sensors: An Example of Space Weather Forecasting by Multiple Satellites

**DOI:** 10.3390/s16040548

**Published:** 2016-04-15

**Authors:** Masahiro Tokumitsu, Keisuke Hasegawa, Yoshiteru Ishida

**Affiliations:** 1 Department of Electrical and Control Engineering, National Institute of Technology, Yonago College, Hikonacho 4448, Yonago 683-0854, Japan; tokumitsu@yonago.kosen-ac.jp; 2 Department of Computer Science and Engineering, Toyohashi University of Technology, Hibarigaoka 1-1, Toyohashi 441-8580, Japan; ishida@cs.tut.ac.jp

**Keywords:** sensor networks, dynamic relational networks, spatiotemporal interpolation, self-recognizing networks, profiling

## Abstract

This paper attempts to construct a resilient sensor network model with an example of space weather forecasting. The proposed model is based on a dynamic relational network. Space weather forecasting is vital for a satellite operation because an operational team needs to make a decision for providing its satellite service. The proposed model is resilient to failures of sensors or missing data due to the satellite operation. In the proposed model, the missing data of a sensor is interpolated by other sensors associated. This paper demonstrates two examples of space weather forecasting that involves the missing observations in some test cases. In these examples, the sensor network for space weather forecasting continues a diagnosis by replacing faulted sensors with virtual ones. The demonstrations showed that the proposed model is resilient against sensor failures due to suspension of hardware failures or technical reasons.

## 1. Introduction

Satellites play an important role in social infrastructure. Satellites provide numerous services: Telecommunications, global positioning system, broadcasting and weather observations and are equipped with sensors. Such sensors, mounted on satellites and spacecrafts could become unavailable due to scheduled satellite operations, technical failures, external disturbances and internal deficiency [1,2]. As a result, failures in sensors can lead to the degraded performance of satellites and their networks including related services as space weather forecasting [2,3].

This paper attempts to extend a dynamic relational network [3,4,5,6] which supports missing of observations. The dynamic relational network is composed of the sensors capable of monitoring particular subjects and processing the data. Each sensor diagnoses other sensors based on relations among them. The faulty sensors are detected by a distributed manner within the dynamic relational network. The dynamic relational network is applied to various kinds of fields: Combustion systems [4], detection of human behaviors [6], space weather forecasting [3] and a plant system [5]. However, the faulty or unavailable sensors affect to a diagnosis of the sensors since some sensors are irresponsible for communications. In the sensor networks, the crucial sensors would fail due to troubles of their electrical circuits or technical reasons. The compromised sensors are needed to be complemented with other surviving sensors.

Numerous studies have tackled construction of resilient computer networks or sensor networks [7,8,9,10,11]. The definition of resilience is discussed in various papers [8] and is different according to their objectives. In these studies, the functions of the missing nodes are recovered or compensated in two ways: replication and complement. For the first approach [10], the functions of the nodes are fully or partially replicated by other nodes. For the second approach [7,11] the functions of the nodes are compensated by actions of other nodes. In the second approach, the networks allow normal sensors to be faulted sensors or malicious states of the sensors, because, their functions are compensated by other nodes.

This paper attempts to compensate the missing data and functions of the faulted nodes in the sensor networks by information processing [12]. The functions of the sensor nodes are virtually created by interactions of neighbor sensors. Further, the missing data of observations by the faulty sensors are also interpolated with information of other sensors. In the proposed model, normal sensors do not require the real sensors for substituting the faulted sensors. In simulations, this paper considers a possibility of the proposed model for the construction of the resilient sensor network. As an example, the demonstration of the proposed model shows a prediction performance for space weather forecasting involving spatiotemporal interpolation. The sensor network for space weather forecasting predicts the high-energy electron flux at geostationary orbit (GEO). The prediction of the high-energy flux at GEO is vital for satellite operation since “hot” (∼keV electron temperatures) plasmas and energetic ( MeV) electrons can lead to electrostatic charging [13] and therefore to satellite failures [1].

The remainder of this paper is organized as follows. Section 2 describes the extension of a dynamic relational network for spatiotemporal interpolation. Further, it also explains profiling statistical properties of observation data by a Support Vector Machine (SVM) [14]. Section 3 examines the performances of the proposed model for missing observations. The sensor network for space weather forecasting is chosen as an example for evaluation. This paper compares simulation results with the observed data. Section 4 discussed a significance of our proposed model and remained issues. Section 5 provides a summary and concludes this paper.

## 2. Extension of Dynamic Relational Network for Spatiotemporal Interpolation

### 2.1. Dynamic Relational Network Model for Space Weather Forecasting

Section 2 extends the dynamic relational network that supports sensor faults by replacing unavailable sensors with virtual sensors. The neighbor sensors virtually create the virtual sensors by communication and information processing, given the fact that actual sensors would be unavailable due to technical reasons or problems of electrical circuits [1]. The virtual sensors could compensate for the faulted sensors, since, the observations can be interpolated by virtual sensors. To this direction, such an extension had not been investigated in the framework of the dynamic relational network. A related study [3] on the dynamic relational network had been proposed. The study reported on the dynamic relational network for space weather forecasting involving virtual sensors for prediction. In that study, the proposed model forecasted the high-energy electron flux at GEO (≥2 MeV), 24 h ahead, while the proposed model, involving virtual sensors, was successful on the prediction of the high-energy electron flux.

This paper proposes the framework for resilient sensor networks based on the dynamic relational network. As an example, we choose a research issue of space weather forecasting for the high-energy electron flux at GEO. The prediction of condition on high-energy electrons at GEO is vital in space weather forecasting. Because, high-energy electrons penetrate into the satellites and charge on the instruments in them. Moreover, high-energy electrons can damage instruments or electrical circuits of the satellites [1,13]. Given the fact that different kind of satellites are in operation at GEO: such as broadcasting, communication, and meteorological satellites, those constitute an essential infrastructure for the society.

A theoretical model for enhancement of high-energy electrons is still under investigation [15]. Some candidate models had been proposed [16,17]. However, a critical evidence for these candidate models has not yet been obtained by satellite observations. The observation of the enhancement of high-energy electrons is preparing in ERG project [18]. This project is going to observe high-energy particles by in-situ sensing with its satellite in 2016.

However, various kinds of models for prediction of high-energy electron flux at GEO have been proposed. The prediction methods are categorized into two categories: Theoretical approach and information-based approach. In the theoretical approach, dynamics on high-energy electrons are forecasted with dynamics and observed data by numerical simulations [17,19,20]. However, the models by this approach are complicated, and it requires many resource for computation (e.g., time and memory). In the information-based approach, on the other hand, various kinds of models have been proposed. For instance, the linear prediction filter has been developed [21] for the prediction of the high-energy electron flux. The studies [22,23,24,25] have also investigated prediction models using neural networks. This paper thinks that the proposed model is categorized into the information-based approach. The relations among sensors are profiled by a knowledge-based information processing. However, space weather forecasting is dynamically predicted by the dynamic relational network model.

This paper demonstrates simulations on space weather forecasting by the proposed model in Section 3. Basically, in the proposed model, the sensor network for space weather forecasting consists of four real sensors and for a single virtual sensor. One of the real sensors is a high-energy electron flux (≥2 MeV) installed on the Geostationary Operational Environmental Satellite 10 (GOES-10) of National Oceanic and Atmospheric Administration. The other real sensors are for the solar wind speed installed on two satellites in a mission of the Solar Terrestrial Relations Observatory (STEREO) [26] and the Advanced Composition Explorer (ACE) [27]. In STEREO mission, STEREO-A and B were launched in 2006 into orbits around the Sun that cause them, respectively, to pull further ahead of and fall gradually behind the Earth. The STEREOs are moving on their orbits, so that the distance between two satellites are longer and longer. On the other hand, ACE were launched in 1997 and is placed at near $L_{1}$ Lagrangian point. The angular separation for STEREO-A, STEREO-B, and ACE, are within $\pm 25^{\circ }$ in 2008. The angular separation of these three satellites are related to prediction performance of the high-energy electron flux.

**Figure 1:**
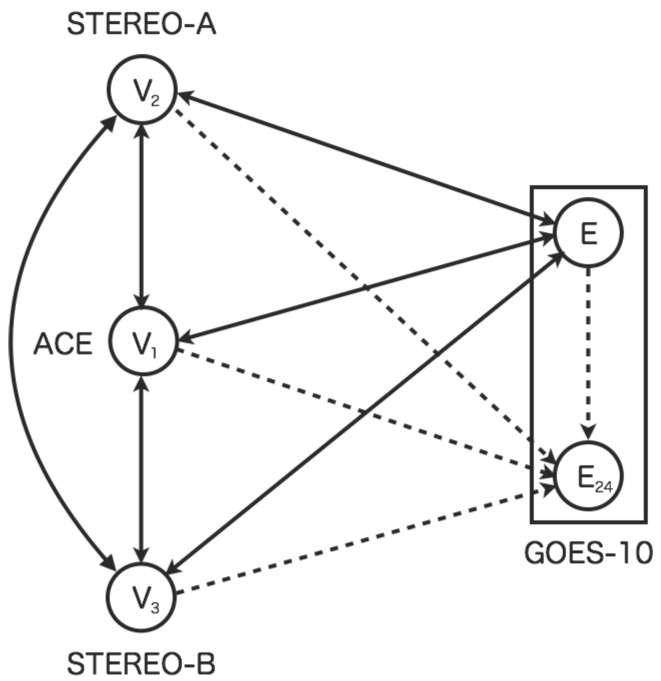
Dynamic relational network for space weather forecast using multiple sensors. The node $V_{i}$ represents the sensor for the solar wind speed. The nodes *E* and $E_{24}$ indicate present high-energy electron flux and flux twenty-four hours ahead. The rectangle represents an internal regions of GOES-10 which equips with couple sensors for observation. Solid line arcs represent a diagnosis flow from a source node to a target node. Dashed line arcs indicate a diagnosis flow involving a prediction from a source node to a target node.

Figure 1 shows the sensor network based on the dynamic relational network. The sensor network consists of the five sensors: four real sensors and one virtual sensor. The sensors of $V_{1}$ (STEREO-A), $V_{2}$ (ACE), and $V_{3}$ (STEREO-B), which represent the solar wind speed. The sensor *E* (GOES-10) represents high-energy electron flux at geostationary orbit. The virtual sensor $E_{24}$ represents the high-energy electron flux 24 h in advance. The real sensors diagnose its state each other. However, the diagnosis by virtual sensors is different. The virtual sensor for prediction does not diagnose other sensors, while the virtual sensors for interpolation do it each other.

In the earlier study [3], the sensor network for space weather forecasting aiming to predict condition on high-energy electrons had been proposed. The proposed model with seven real sensors and single virtual sensor: Three sensors for the solar wind speed, three sensors for north-south component of interplanetary magnetic field (IMF) and the high-energy electron flux and its flux 24 h ahead. However, this paper chooses the four real sensors and the single virtual sensor for space weather forecasting: Three sensors for the solar wind speed, the single sensor for the high-energy electron flux and its flux 24 h ahead. The north-south component of IMF is also the crucial parameter for detecting precursor of changing of the space environment around the Earth. The proposed model does not involve the three real sensors of IMF, because that complicates the model. Therefore, this paper keeps the simplicity of the proposed model so that we are able to consider the main aim of possibility for spatiotemporal interpolation in sensor networks.

### 2.2. Dynamics of Dynamic Relational Network Model

For computation, three kinds of representative models had been proposed for a dynamic relational network [5]: Black and white model, skeptical model, and gray model. The basic model is black and white model. This paper uses the gray model for the simulations.

In the dynamic relational network, the sensor status is represented by a credibility: normal (credibility = 1) or faulty (credibility = 0). This paper uses $R_{i}$ to denote a credibility of a sensor node *i*. The credibility is a time variable so that the value will be changed by mutual evaluation according to a time development. We denote the relationship between two sensors as $T_{ij}$. The value of the parameter $T_{ji}$ is determined by the diagnosis from the source node *i* to the target node *j*. The source node *i* evaluates the target node *j* based on their relation. An arc represents the relation between two sensors described as profiles using the SVM.

For the black and white model, the dynamics of the dynamic relational network are described as follows:

(1) $$\frac{dr_{i}\left(t\right)}{dt}=\sum_{j}T_{ji}R_{j}+\sum_{j}T_{ij}R_{j}-\frac{1}{2}\sum_{i}\left(T_{ij}+1\right)$$

where

(2) $$R_{i}\left(t\right)=\frac{1}{1+exp(-r_{i}\left(t\right))}$$

$r_{i}\left(t\right)\in \left(-\infty ,+\infty \right)$ is a time variable which represents an accumulation of a diagnosis result. Equation (1) describes the dynamics of the credibility for a sensor node i. Equation (2) defines a mapping function of $r_{i}\left(t\right)$ to $R_{i}\left(t\right)\in \left[0,1\right]$. The time variable $r_{i}$ is mapped into the credibility $R_{i}$ through Equation (2). The convergence of the credibilities are theoretically guaranteed in the black and white model. The credibilities are converged to 0 or 1.

In the gray model, the credibility can be an intermediate value between 0 and 1. The credibility of the gray model can reflect ambiguous states that do not determine exactly whether the status of the sensors is normal or abnormal. Space weather forecasting of high-energy electron flux also involves ambiguous situations by the change of unpredictable flux. With the gray model, the risk for changes of high-energy electron flux can be considered by the credibility.

(3) $$\frac{dr_{i}\left(t\right)}{dt}=\sum_{j}T_{ji}^{+}R_{j}\left(t\right)-r_{i}\left(t\right)$$

where

(4) $$\begin{array}{ccc} T_{ji}^{+} & = & \left\{\begin{array}{cc} T_{ij}+T_{ji}-1 & \left(\mathrm{if}\;\mathrm{there}\;\mathrm{are}\;\mathrm{arcs}\;\mathrm{between}\;i\;\mathrm{and}\;j)\right)\\ 0 & \left(\mathrm{if}\;\mathrm{there}\;\mathrm{no}\;\mathrm{arc}\;\mathrm{between}\;i\;\mathrm{and}\;j\right) \end{array}\right. \end{array}$$

In this model, the time variable $r_{i}\left(t\right)$ is also mapped into the credibility $R_{i}$ by Equation (2).

### 2.3. Modeling of Relations between Two Sensors

This paper involves statistical properties of the observation data using the SVM [14]. The proposed method involves not only profiling of the observed data [3] but also spatiotemporal interpolation of the missing data. The SVM is a superior method in machine learning for classifying input data into two categories: +1 and -1. The sensor nodes appropriately evaluate the sensor status based on the relation between two sensors, so that the SVM classifies the input data nonlinearly into two categories. Further, the SVM also can be used for regression of the data. Therefore, this paper selects the SVM as a profiling method for estimating relations and interpolating data between two sensors.

**Figure 2:**
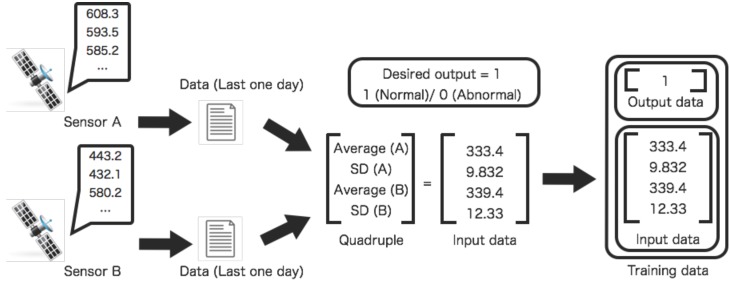
Training data generation process for two sensors. The training data of the sensors are created from the observed data. The training data consists of averaged values, their standard deviations, and the desired output.

The profiles correspond to relations between two sensors. The profiles are described by parameters of the SVM. The profiles are created by training of the specified data set. The training input data consists of a quadruple and a desired output value (Figure 2). The quadruple consists of four values: averaged values for the past one day and their standard deviations. The averaged values and their standard deviations are calculated from a day in the past. In other words, twenty-four points of observations are used for making a data of the training data set. The desired output value can be binary values: Normal (0) and abnormal (1). The desired output value is determined by classifying conditions of the high-energy electron fluxes. The high-energy electron flux is also categorised into binary values: Quiet (−1) and alert (1) levels. The training data set for an abnormal condition, solar-wind speed and high-energy electron flux, are chosen from the data set corresponding to the condition of an alert level flux. In this paper, the level of the high-energy electron fluxes are quantified by a threshold. The condition of the high-energy electron fluxes are identified as normal where the flux level is less than the threshold. The threshold of classifying condition on high-energy electron flux is 3.0. This criterion is used in the space weather forecasting [28].

**Figure 3:**
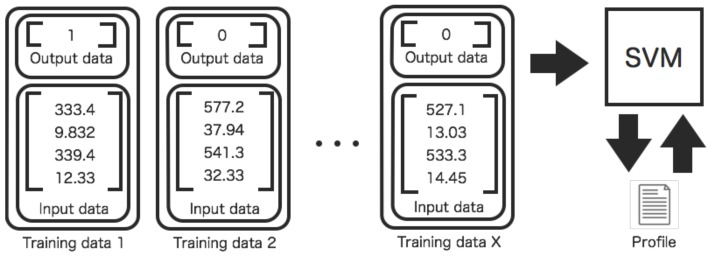
Training process of a profile between two sensors. The training data set are input to an SVM. The SVM learns desired outputs from the training data set.

**Figure 4:**
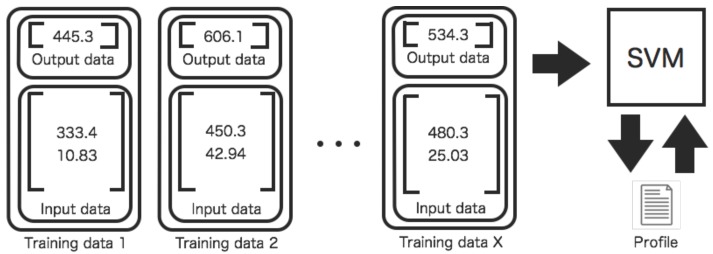
Training process of a profile between two sensors for spatiotemporal interpolation of missing data. The training data consisting of tuple data set are input to an SVM. The SVM learns desired outputs (interpolated value) from the training data set. This profile is used for interpolation. On the other hand, the profile shown in Figure 3 is used for evaluating relations between two sensors.

Two kinds of profiles are created in order to evaluate and interpolate relations among sensors (Figure 3 and Figure 4). For the former one, the profiles are used for evaluating relations between two sensors. For the latter one, the profiles are used for interpolating missing data between two sensors. The training process of profiles is different. The parameters of the SVM are repeatedly adjusted to make desired outputs by training input data.

For interpolation of the missing data, the sensor nodes try to complement the faulted sensors. The faulted sensors are replaced with virtual sensors by actions of neighbor nodes. Eventually, the missing data in observations are interpolated by neighbor nodes. The missing data of observations are finalized by averaging interpolated values. The evaluations of the relations are diagnosed between two sensors in the dynamic relational network.

Figure 4 shows the generation process for profiles aiming to interpolate missing data. The quadruple data for the test between two sensors is unavailable if either one of two sensors is faulted. Therefore, a tuple of the data composed of only available sensor is used for interpolation of missing data. The input data for interpolation By interpolation of missing data, the test process for the sensors is also applicable without modifying the process for quadruple data.

### 2.4. Tests of Relations in Dynamic Relational Network

**Figure 5:**
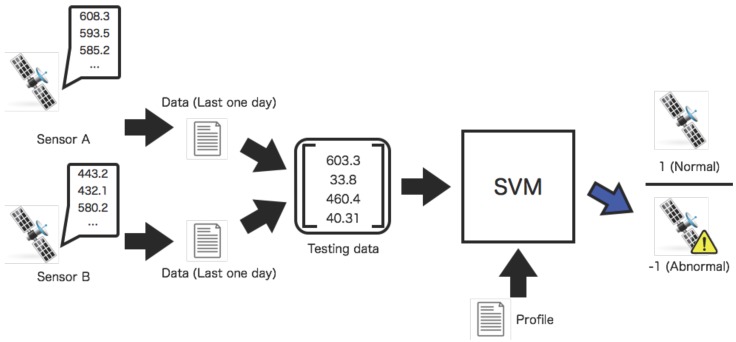
Test of observed data recorded by two sensors. The test data are input to an SVM based on the profile. The SVM classifies the condition of the space environment as binary values 1 (normal) or −1 (abnormal).

In the test phase, the diagnosis of the relation between two sensors is executed with two sensor values shown in Figure 5. Similar to the training phase, the input data for the test is comprised using the values of the two sensors that are observed values are averaged a day in the past. The test data for the diagnosis is input to the SVM, which represents the relation between two sensors. The SVM outputs the result whether the test data satisfies the predefined conditions of the space environment. The SVM outputs 1 where the input data for the test is contained in the profile at the quiet level of the high-energy electron flux, otherwise −1.

**Figure 6:**
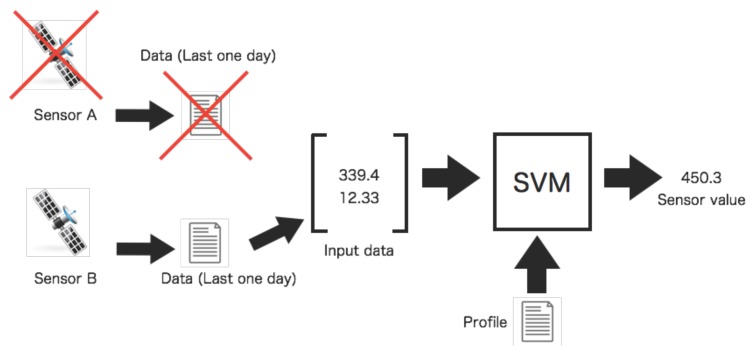
Interpolation process of missing data. The interpolation of the missing data is calculated by the data last one day of the opposite side. The interpolated value is calculated by the SVM with the input data and the predefined profile.

Before the diagnosis, however, the quadruple data for tests is not always available. The missing data is interpolated by two sensors associated by using the SVM if the observation data is lacking. The interpolated data is calculated by using the SVM with the data observed by the single sensor. In Figure 6, the missing data of Sensor A is interpolated by the value of Sensor B using the SVM with the predefined profile. In this case, the missing data of Sensor A is necessary for interpolation since Sensor A is faulty. The tests of relations among sensors are executed after interpolating missing data.

**Figure 7:**
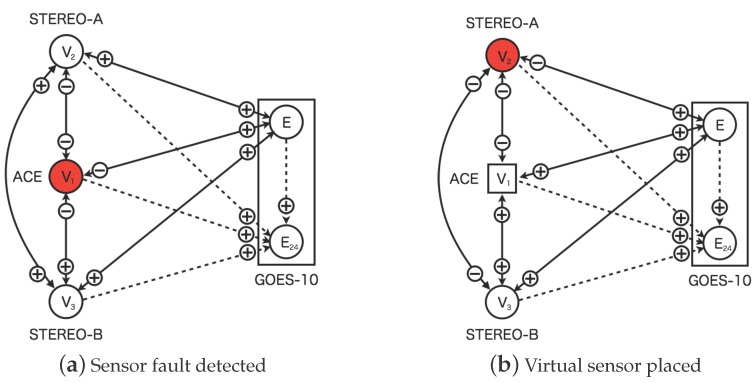
Replacement process of faulted sensors in the dynamic relational network. (**a**) The real sensor ($V_{1}$) of ACE is detected as fault; (**b**) The virtual sensor ($V_{1}$) of ACE is created. The circles and squares indicate the real sensor nodes and the virtual sensor nodes respectively. The sensor nodes colored white (colored red) are normal (abnormal). The rectangles indicate region of the satellite. The two sensors of GOES-10 is shown in the regions of the rectangles. The solid lines indicate diagnosis flow of mutual diagnosis. The dashed line indicate diagnosis flow from real sensors to virtual sensors for prediction. The plus (minus) indicate diagnosis result normal (abnormal) from source nodes to target nodes.

Figure 7 shows the replacement process of the faulted sensor in the dynamic relational network. In the proposed model, the sensors are diagnosed from other sensors connected. The neighbor sensors would identify the faulted sensors as abnormal by the distributed manner. The faulted sensor is disjointed from the dynamic relational network where the sensor status identified as abnormal for 24 h continuously. In Figure 7a, the solar-wind sensor of ACE is diagnosed as abnormal from other three nodes. In Figure 7b on the other hand, the solar wind sensor of ACE is replaced a circle node to rectangle one. The rectangle node indicates the virtual sensor behaving as virtual sensor which is created from information of neighbor nodes. The virtual sensor is also diagnosed from other sensor nodes.

**Figure 8:**
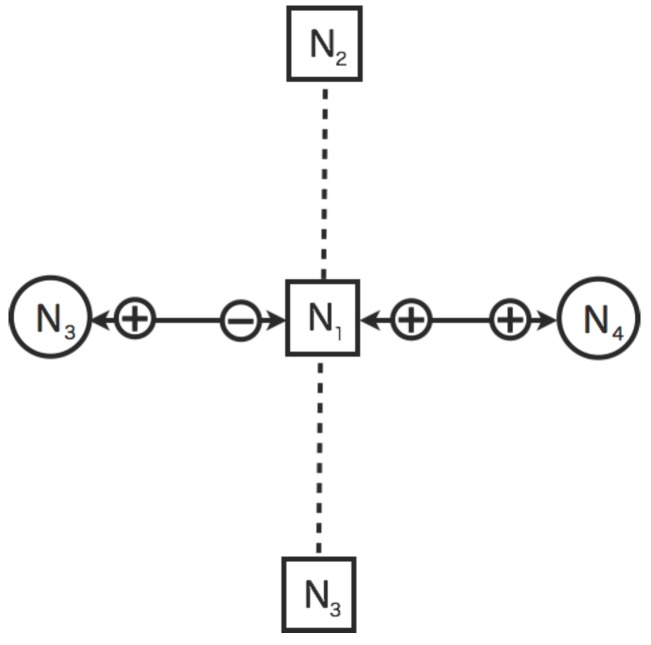
An illustrative example of a constraint on virtual sensors in the dynamic relational network. The circles and squares indicate the real and virtual sensor nodes respectively. The sensor nodes colored white is normal. The solid lines indicate diagnosis flow of mutual diagnosis. The dashed line indicate diagnosis flow from real sensors to virtual sensors. The plus (minus) indicate the diagnosis result as normal (abnormal) from the source node to the target node.

In Figure 8, the virtual sensors are constrained to diagnose from the neighbor nodes. The interpolated value of the virtual sensor is not reliable since it is the result that are accumulated sensor values by the neighbor nodes. In the proposed model, the diagnosis between virtual sensors for interpolation is not allowed. The virtual sensors $N_{1}$ and $N_{2}$ ($N_{3}$) are not allowed to diagnosis each other. Because the virtual sensors simultaneously need to get the data from each other at that time. The sensor value of one sensor depends on the data of another one. Therefore, the sensor value of the virtual sensor interpolated by another virtual one is not determined simultaneously. However, the virtual sensors for prediction and interpolation are allowed to diagnosis each other.

## 3. Simulations

This section examines a significance of the proposed model by the tests with the actual observed data. This paper considers two cases: (1) a fault of a single sensor; (2) faults of multiple (two) sensors. The results of the demonstrations for the test data show plots of the time development of the credibility for each sensor. The demonstrations also compare with the actual and predicted data of the high-energy electron flux. Further, this paper considers the comparisons of averaged credibilities for the test data set.

### 3.1. Data Description

This paper uses solar wind speed data observed by different three satellites: ACE, STEREO-A, and STEREO-B. The solar wind data observed by STEREO-A and STEREO-B are obtained from the Coordinated Data Analysis Web (CDAWeb) [29] in the National Space Science Data Center (NSSDC) of the National Aeronautics and Space Administration/Goddard Space Flight Center (NASA/GSFC). The solar wind data observed by the Advanced Composition Explorer (ACE) satellite are obtained from the OMNI-2 database [30] in the National Space Science Data Center (NSSDC) of the National Aeronautics and Space Administration/Goddard Space Flight Center (NASA/GSFC). The electron flux data observed by the GOES-10 satellite are obtained from the National Geophysical Data Center (NGDC) and the National Oceanic and Atmospheric Administration (NOAA/NGDC) [28].

This paper obtained all data during the period from 2 March 2007 to 7 December 2009, approximately two years in total. The period of the training data set is from 2 March 2007 to 14 December 2007. The training data set is used to generate profiles among the sensors that are parameters for the SVM. For each sensor, the total number of the training sequences is 4457, that are generated from the actual observed data. This paper excludes the missing data from the training data set. This paper defines the missing data if the missing exceeds three hours. The other missing data, is less than or equal three hours, are compensated with a spline interpolation.

The total number of the training sequences for the sensor faults is 6950, which involves the data for the fault of the single sensor between two sensors. This paper replaced the actual data with abnormal data. Because the abnormal data corresponding to the sensor failure is not involved in the observations. The abnormal data is needed to be generated artificially by hand. The abnormal data represents faulted status of the sensors generated by averaged values of observations and their standard deviations. The range of the abnormal data is from 0.0 to absolute values discounted by 0.1 of the minimum observed value. The abnormal data is randomly generated with a uniform distribution and replaced the normal data with their values in a generation of the test data sequences.

**Figure 9:**
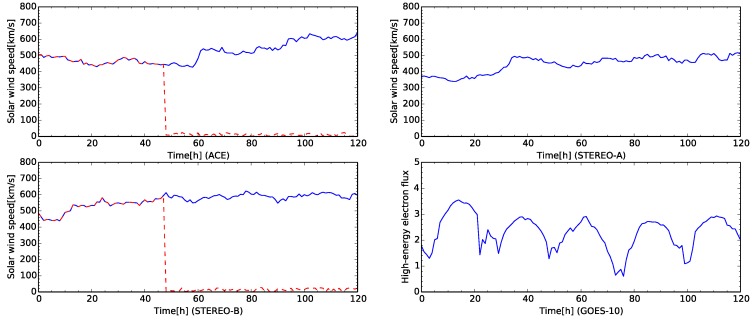
Time development of the test case for five days from 1 May 2008 at 00:00:00 UTC. The satellite names are labeled in horizontal axes. The plots in the first row and left one in the second row show the time development of solar wind speed. The right plot in the second row shows the time development of the high-energy electron flux. The solar wind plots for ACE and STEREO-B also show the abnormal data for the test (dashed line).

Figure 9 shows the time development of the observed data for five days from 1 May 2008 at 00:00:00 UTC. In Figure 9, the solar wind speed of ACE is about 500 km/s for 60 h from the beginning of the plot. The solar wind speed of ACE rapidly increased from about 500 km/s to 600 km/s. The solar wind speed of STEREO-A is about 400 km/s for 30 h from the beginning of the plot. After that, the solar wind speed of STEREO-A also rapidly increases, then it keeps the speed about 500 km/s. The solar wind speed of STEREO-B also increases to about 600 km/s after 10 h from the beginning of the time development. The high-energy electron flux at the geostationary orbit of GOES-10 keeps at the quiet level because the flux level is less than 4. In Figure 9, the daily variations of the high-energy electron flux are also observed. The flux level varies from 1 to approximately 4.

The test data for the simulation consists of values observed by four sensors. For the test, sensor fault data is artificially created by hand. The sensor data actually observed is shown a solid line in Figure 9. In the case studies, the sensors for the solar wind speed of ACE and STEREO-A satellites are faulted in the data at 48 from the beginning in the test sequence shown as a dashed line in Figure 9.

### 3.2. Case Study 1: Fault of Single Sensor

**Figure 10:**
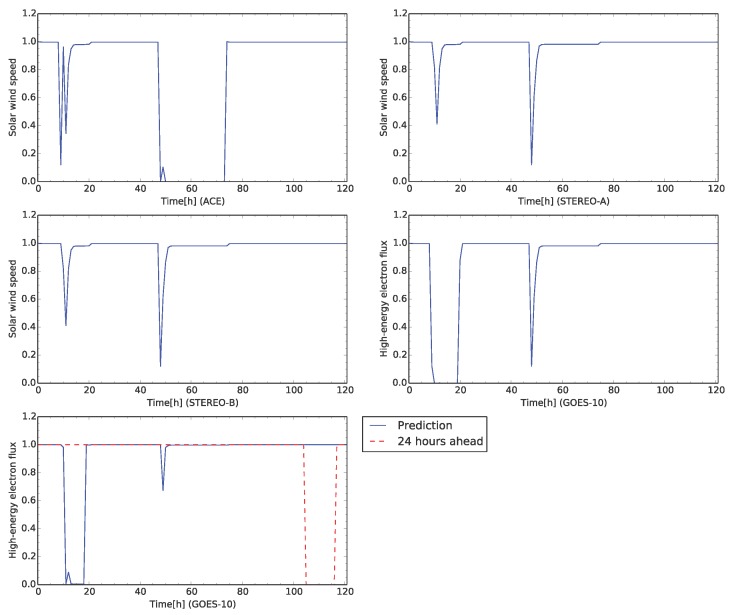
Time development of the credibility of each sensor for the test case shown in Figure 9. The satellite names are labeled in horizontal axes. The plots in the first row and the left one in the second row show the time development of the credibility for the solar wind speed. The right plot in the second row (in the third row) show the time development of the high-energy electron flux (high-energy electron flux 24 h ahead).

In this case study, the sensor of ACE for the solar wind speed is faulted at 48 h from the beginning of the time development. Figure 10 shows the time development for the test case. The credibility of the solar wind speed observed by ACE, STEREO-A, and STEREO-B show rapidly decrease to less than 0.4 at the beginning of the simulation. Because the sensors of the solar wind speed also detected its rapid increase. The high-energy electron flux also decreases to small values. This change of the credibility corresponds to rapid increases of the high-energy electron flux and the solar wind speed observed by STEREO-B because the flux level grows to around 4.0 of the alarm level [31]. During this change, the virtual sensors for interpolation is not appeared.

**Figure 11:**
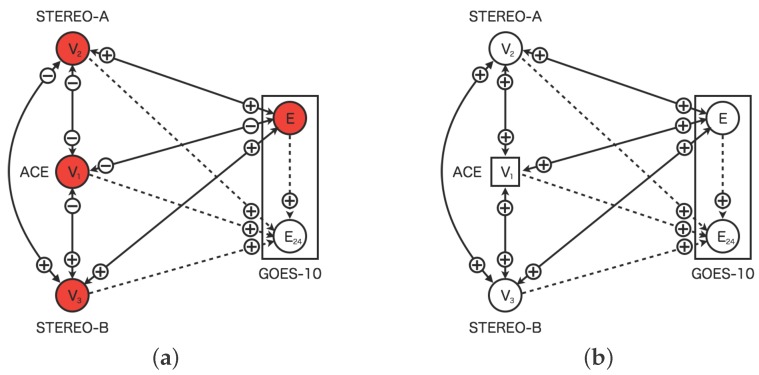
Network snapshot of the dynamic relational network for the test data shown in Figure 9. Figure (**a**,**b**) show status of the nodes at 48 and 80 steps respectively. The circles and squares indicate the real and virtual sensor nodes respectively. The white (red) colored sensor nodes is normal (abnormal). The credibility for normal status is larger than 0.5, otherwise abnormal. The rectangles indicate region of the satellite. The two sensors of GOES-10 is shown in the regions of the rectangles. The solid lines indicate diagnosis flow of mutual diagnosis. The dashed line indicate diagnosis flow from real sensors to virtual sensors for prediction. The marks plus (minus) indicate diagnosis result normal (abnormal) from source nodes to target nodes. (**a**) Step = 48 (After the real sensor for ACE failed); (**b**) Step = 80 (After the virtual sensor for ACE is created).

Further, Figure 10 shows the same change of the credibility for the sensors of the solar wind speed. The sensor for the solar wind speed of ACE keeps the very small credibility because the sensor is faulted at 48 h from the beginning. Figure 11 shows network snapshots for the test case shown in Figure 9. The sensor of ACE is identified as abnormal by other sensors associated in Figure 11a. The signs of all arcs to ACE show minus which means the observed value of ACE deviated from the predefined profile. The sensors of STEREO-A, STEREO-B and GOES-10 also show abnormal status. However, the credibility of STEREO-A, STEREO-B, and GOES-10 grows to 1.0 again shortly.

The credibility of the solar wind speed of ACE keeps to zero to 72 h from the beginning of the time development. The sensor fault is identified if the credibility keeps the threshold for 24 h. In this test case, the sensors which are connected to the solar wind speed ACE, identify it as fault after 24 h later the failure occurs. After detecting sensor faults, the solar wind speed of ACE is replaced by the virtual sensor and its credibility is returned to 1 again. The credibility of the sensors keeps at 1.0 after the virtual sensor for the solar wind speed of ACE appears. All sensors are normal at 80 h from the beginning of the time development in Figure 11b. The time, 80 h from the beginning, corresponds to the recovery of the sensor fault of ACE.

The sensors of the high-energy electron flux and its flux 24 h ahead, the credibilities of both sensors show different between observed data and prediction in Figure 10. The possible reason of this difference is due to replacement of the virtual sensor instead of the actual sensor. The sensor value of the virtual sensor is interpolated by the associated sensors. The interpolated sensor value is averaged values of the estimated values from the neighbor sensors, and the averaged value is interpolated value, not the observed value. In this test case, the solar wind speed of ACE gradually increases at 90 h from the beginning. The virtual sensor trained with the data set is not able to follow this change of the solar wind speed. However, the simulation shows the different rate between observed and predicted data is 0.20. The prediction and interpolation of the virtual sensors are successful in this test case.

### 3.3. Case Study 2: Faults of Multiple Sensors

**Figure 12:**
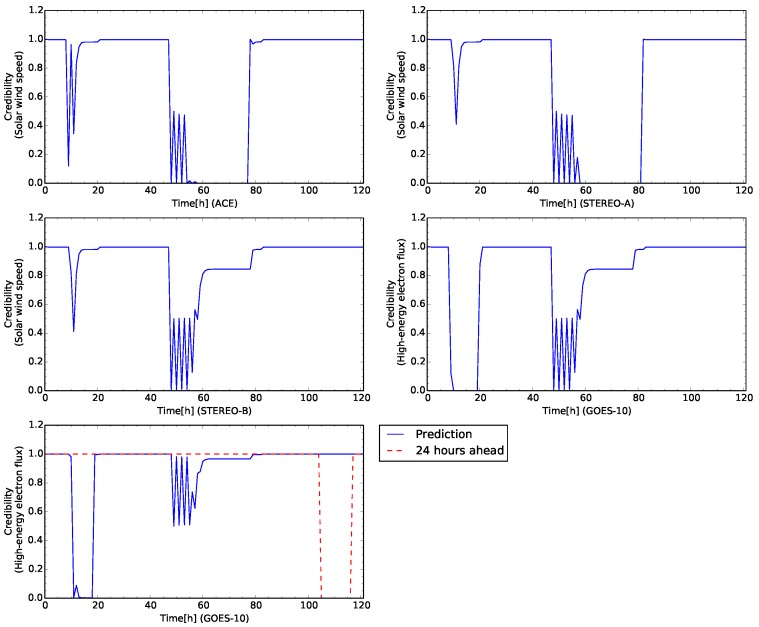
Time development of the credibility of each sensor for the test case shown in Figure 9. The satellite names are labeled in horizontal axes. The plots in the first row and the left one in the second row show the time development of the credibility for the solar wind speed. The right plot in the second row (in the third row) show the time development of the high-energy electron flux (high-energy electron flux 24 h ahead).

**Figure 13:**
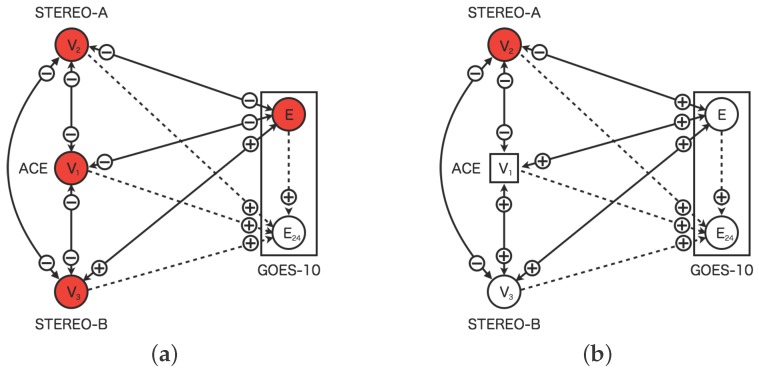
Network snapshot of the dynamic relational network for the test data shown in Figure 9. Figure (**a**,**b**) show the status of the nodes at 48 and 80 steps respectively. The circles and squares indicate the real and virtual sensor nodes respectively. The white (red) colored sensor nodes is normal (abnormal). The credibility for the normal status is larger than 0.5, otherwise abnormal. The rectangles indicate region of the satellite. The two sensors of GOES-10 is shown in the regions of the rectangles. The solid lines indicate the diagnosis flows of mutual diagnosis. The dashed line indicate diagnosis flows from real sensors to virtual sensors for prediction. The marks plus (minus) indicate diagnosis results as normal (abnormal) from the source nodes to the target nodes. (**a**) Step = 48 (After the real sensor for ACE failed); (**b**) Step = 80 (After the virtual sensor for ACE is created).

In this case study, the sensors of ACE and STEREO-A for the solar wind speed are faulted at 48 h from the beginning of the time development. The both sensors fail simultaneously in the test data. Figure 12 shows the time development for the test case, however, this case involves the faults of the two sensors. The time development for all sensors is the same up to 48 h from the start of the test sequence. The two sensors of ACE and STEREO-A fail at 48 h from the beginning of the time development. Figure 13 shows network snapshots for the test case involving the faults of the two sensors shown in Figure 9. The sensors of ACE and STEREO-A are identified as abnormal by the other sensors associated in Figure 13b. After detection of the sensors faults, all plots of the credibility oscillates between 0.0 and 0.5.

For the sensor of STEREO-A, the oscillation period of the credibility is longer than that one of ACE. After oscillation is stable, the plots of the credibility for the sensors of ACE and STEREO-A keep to zero until about 80 h from the beginning of the time development. For ACE, the credibility of the sensor recovered to 1.0 at 78 h. After this recovery, For STEREO-A, the credibility of the sensors also recovered to 1.0 at 82 h. This delay is caused by the oscillation period of the credibility when the sensor faults happen.

The faulted sensor of ACE is replaced with the virtual sensor at 80 h shown in Figure 13b, and after 24 h both sensors are identified as abnormal. However, the sensor of STEREO-A is still the real sensor because the virtual sensor is created after it is identified as abnormal in 24 h. In Figure 12 the period of the oscillation for the credibility is longer than the value of ACE. At 82 h, the credibility of ACE is changed to 1.0 by replacing with the virtual sensor.

After two virtual sensors are created, in Figure 12, the plots of the time development is similar to the Case Study 1 in Section 3.2. The credibility of the high-energy electron flux is not successful though the flux level attains to the alert level in the observed data.

### 3.4. Significance of Spatiotemporal Interpolation in Space Weather Forecasting

This paper compares credibilities of the sensors whether the spatiotemporal interpolation of the missing sensors is significant. The credibility is calculated from the time sequences of the simulation results after 72 h from the start. The time 72 h is minimum time that the faulted sensors are replaced with virtual ones by the neighbor sensors. The total number of the test sequences is 9907.

Table 1 shows comparisons of the averaged credibility for each sensor for both the Case Study 1 (Section 3.2) and the Case Study 2 (Section 3.3). The sensor of ACE fails in Case Study 1. In Table 1a, the credibilities with the spatiotemporal interpolation are larger than without it. The credibility with the spatiotemporal interpolation is 0.8 while without one is about 0.0. The faulted sensor is replaced with virtual one by the point of view of the credibility. In Table 1b, on the other hand, the credibilities for both sensors of ACE and STEREO-A are larger than that ones without the interpolation. With the spatiotemporal interpolation, the credibilities of both sensors are 0.6, while about 0.0. In this case, the faulted sensors are also replaced with virtual ones by the point of view of the credibility. Therefore, generating virtual sensors by the spatiotemporal interpolation is significant in these two case studies.

Table 2 shows a performance comparison of forecasting for the high-energy electron flux 24 h ahead. This paper compares a prediction success rate as a performance measure. This paper defines the prediction success rate as a fraction of warning alarm of the high-energy electron flux 24 h ahead. The sensor $E_{24}$ alarms if its credibility is less than 0.5. The prediction performances for both cases of the single and the two sensors failed do not show any significant difference. For the case of the single sensor failed, the prediction success rates (used or unused) are almost equal in both cases. For the case of the two sensor failed, furthermore, the prediction success rates (used or unused) are equal in both cases. In these case studies, single or multiple faults of the sensors do not affect to the statistical performance for prediction of the high-energy electron flux 24 h ahead.

In some test cases, however, the sensor faults are recovered by the mutual diagnosis and the sensor replacement. Moreover, the credibilities of the sensors with spatiotemporal interpolation are larger than without it. Therefore, the spatiotemporal interpolation for faulted sensors is a significant approach to keep the system reliability, because the credibilities of some sensors are recovered as shown in Table 1.

## 4. Discussion

This paper extended the dynamic relational network involving spatiotemporal interpolation of the missing data of the sensors. In the earlier study [3], the dynamic relational network includes real and virtual sensors. The virtual sensors in the previous models are defined for prediction. In this paper, however, the virtual sensors are defined for prediction and interpolation of the missing data. The proposed model [12] of this work handles the sensor faults and compensates the missing data using the sensors associated.

The simulations of this paper demonstrated that the missing data of the faulted sensors are interpolated by averaging observed values of the neighbor sensors. The sensor value estimated by the single sensor is not plausible. However the averaged sensor values are interpolated from the several sensors. This paper examined two scenarios as the case studies: The fault of the single sensor (Section 3.2) and faults of the multiple sensors (Section 3.3).

In Case Study 1, the single sensor fails, however, the faulted sensor was replaced with the virtual sensor by information processing. In the simulation, finally, the virtual sensor compensates the real one that outputs the interpolated values estimated by the neighbor sensors. In Case Study 2, further, neighbor sensors were simultaneously unsuccessful to create virtual ones in the demonstration. Finally, however, the both sensors were also replaced with virtual ones. This paper also showed the significance of the proposed model in Table 1 because the credibilities with the spatiotemporal interpolation are larger than without one. The spatiotemporal interpolation by neighbor nodes for the virtual sensors support the resilience of the network. However, the period until replacing with virtual sensors depends on the observed data. The plots on the credibility of the time development showed that the credibilities of the sensors oscillated between small and intermediate values in Figure 12.

Constructing the resilient sensor networks is an important issue in computer science. Various kinds of studies have attempted to build the resilient computer networks and sensor networks [7,8,9,10,11]. In these studies, the functions of the missing nodes are recovered or compensated in two ways: replication and complement. The proposed approach of this paper belongs to the complement of malicious states of the nodes. In our approach, the faulted sensors are replaced with virtual ones by information processing. The proposed approach creates the virtual sensors as if they had been in the sensor network. In the case studies, the proposed model detects the faulty sensors and create virtual ones. Finally, space weather forecasting based on the dynamic relational network was successful, even the real sensors fail in operation. Therefore, the spatiotemporal interpolation for missing sensors is the significant approach for achieving the resilient sensor networks.

In the proposed model, resilience for faulted sensors depend on various kinds of properties such as network structures, number of faulty sensors, locations of the sensors, and relations among sensors. The precision of spatiotemporal interpolation depend on relations among the sensors. In this study, for instance, the locations of STEREO-A and STEREO-B are changing, so that they are moving on their orbit. Their relations for ACE are also changing dynamically. This paper selected the data set in 2008 for training the profiles. However, the period of the test data set covers from 2008 to 2009. In other words, the relations need to involve the changes of the sensor locations by updating the profiles among them.

An other example is the number of the nodes in the sensor networks. Some sensor nodes require a few nodes for spatiotemporal interpolation if they had been faulty. On the other hand, some faulted nodes need many neighbor sensors for creating virtual sensors. These characteristics would be found by analysing relations among sensors. In other case, resilience of the sensor networks would vanish if the number of the faulty nodes had exceeded the particular threshold. Identifying relations among sensors is not only crucial topic but also it would be helpful information for constructing the resilient sensor networks.

This paper states there are remained issues to build information systems based on the proposed model. Profiling relations among sensors is also a crucial topic to obtain resilience of the sensor networks. This paper uses the SVM to describe relations among the sensors as profiles. The SVM-based profiles were used for diagnosis of relations and interpolation of the missing data. The interpolation of the sensor values between two sensors involves an effect of time series data since the input data for the SVM is a sequence the past 24 h. Modeling and profiling the relations among the sensors depend on the data processing. The work of this paper is the first step for constructing the resilient sensor networks that handle the missing sensors. Other techniques for creating input data and interpolation methods are available and applicable for the relationships among the sensors, such as Hidden Markov Models (HMMs) and Vector Autoregressive Models (VAR models) [4,6].

This paper considered a computation model to attain the resilient sensor networks with information processing. Protocols for communications among sensors are also necessary to build applications of the proposed model. The sensors need to exchange their observed data and information among them by following the protocols. Concrete examples for the protocols are ZigBee [32] and Simple Network Management Protocol (SNMP) [33]. In computer networks, further, the Internet of Things (IoT) is spreading to collect information from the real world. The resilience of the sensor networks is the crucial topic because the actuators (e.g., satellites) in the networks depend on each other. Therefore, the protocol involving the idea of the proposed model is also required to construct the resilient sensor networks.

## 5. Conclusions

This paper proposed the resilient sensor network model based on the dynamic relational network. The proposed model supports missing of observation by the spatiotemporal interpolation. Further, the proposed model also involves the virtual sensors that are capable of prediction and interpolation. In the proposed model, the missing data is interpolated by the sensors associated with the predefined profiles among them. This paper demonstrated two case studies: the fault of the single sensor and the faults of the multiple sensors. These case studies are for space weather forecasting involving the missing of observation. In both case studies, the sensor network for space weather forecasting continued a diagnosis by replacing the faulted sensor with virtual ones. The demonstrations showed that the proposed model is resilient against sensor failures due to the suspension of the hardware failure or the technical reasons of satellite operations.

## Figures and Tables

**Table 1:** Comparison of the credibility for each sensor. Each cell indicates the credibility. The sensors $V_{1}$, $V_{2}$, and $V_{3}$ respectively correspond to the solar wind speed of ACE, STEREO-A, and STEREO-B. The sensors *E* and $E_{24}$ respectively correspond to the high-energy electron flux and its flux 24 h ahead. Case study 1 (Fault of a single sensor). The sensor of ACE for the solar wind speed is faulted. (**a**) Case study 1 (Fault of a single sensor). The sensor of ACE for the solar wind speed is faulted; (**b**) Case study 2 (Faults of two sensors). The sensors of ACE and STEREO-A for the solar wind speed failed.

**(a)**					
	E	$V_{1}$	$V_{2}$	$V_{3}$	$E_{24}$
**Interpolation (Used)**	0.795	0.803	0.884	0.981	0.8501
**Interpolaton (Unused)**	0.793	0.022	0.821	0.826	0.8081
(**b**)					
	E	$V_{1}$	$V_{2}$	$V_{3}$	$E_{24}$
**Interpolation (Used)**	0.739	0.631	0.627	0.775	0.8181
**Interpolation (Unused)**	0.630	0.048	0.048	0.637	0.7691

**Table 2:** Performance comparison of forecasting for high-energy electron flux 24 h ahead. Each cell in the table shows a prediction success rate where the credibility threshold is 0.5. The sensor $E_{24}$ alarms if its credibility is less than or equal the threshold.

	Single Sensor Failed	Two Sensors Failed
**Interpolation (Used)**	0.849	0.833
**Interpolation (Unused)**	0.850	0.834
